# Structure and function of HCV IRES domains

**DOI:** 10.1016/j.virusres.2008.06.004

**Published:** 2009-02

**Authors:** Peter J. Lukavsky

**Affiliations:** Laboratory of Molecular Biology, MRC, Hills Road, Cambridge CB2 0QH, UK

**Keywords:** Hepatitis C virus (HCV), Internal ribosome entry site (IRES), NMR spectroscopy, RNA structure

## Abstract

The HCV IRES is a highly structured RNA which mediates cap-independent translation initiation in higher eukaryotes. This function is encoded in conserved structural motifs in the two major domains of HCV and HCV-like IRESs, which play crucial and distinct roles along the initiation pathway. In this review, I discuss structural features of IRES domains and how these RNA motifs function as RNA-based initiation factors to form 48S initiation complexes and 80S ribosomes with only a subset of canonical, protein-based eukaryotic initiation factors.

## 1 Introduction

Eukaryotic translation initiation is usually a protein-mediated process, which requires the full complement of canonical eukaryotic initiation factors (eIFs) and a 5′-capped mRNA (Hershey and Merrick, 2000; Kapp and Lorsch, 2004; Merrick, 2004; Sachs et al., 1997). The 80S ribosome assembly starts with recognition of the 5′-cap structure by the eIF4F complex, consisting of eIF4E (cap-binding protein), eIF4A (RNA helicase) and the scaffold protein, eIF4G. This complex in turn recruits the 43S pre-initiation complex comprising the 40S small ribosomal subunit, eIF1, 1A, 3 and the $\text{eIF2}/\text{GTP}/\text{Met-tRN}{\text{A}}_{\text{i}}^{\text{Met}}$ ternary complex to the 5′ end of the mRNA. Subsequently, the ribosomal assembly scans the 5′ UTR of the mRNA with the help of helicase eIF4A and its co-factor eIF4B. During scanning, the 40S ribosome is believed to adopt an open conformation introduced by the binding of eIF1A in the ribosomal A site and eIF1 near the platform (Passmore et al., 2007). Binding of the latter also serves to inhibit hydrolysis of eIF2-bound GTP until the start codon is reached (Algire et al., 2005; Unbehaun et al., 2004). Upon AUG start codon recognition, codon–anticodon base pairing between the mRNA and $\text{Met-tRNA}{\text{i}}_{\text{i}}^{\text{Met}}$ in the ribosomal P site triggers eIF1 release and thereby eIF5-mediated hydrolysis of eIF2-bound GTP (Algire et al., 2005; Unbehaun et al., 2004). Subsequent dissociation of eIF2/GDP and eIF3 from the 48S complex requires eIF5B and the joining of the 60S subunit in another GTP-dependent process to form elongation-competent 80S ribosomes (Algire et al., 2005; Pestova et al., 2000; Pisarev et al., 2006; Unbehaun et al., 2004).

In the translation initiation pathway mediated by HCV and HCV-like IRES RNAs, 48S complex formation does not require a 5′-cap structure or scanning, and only a small subset of the canonical eIFs is sufficient (Hellen and Sarnow, 2001; Sachs et al., 1997). Instead of being mediated by external protein factors (eIFs), HCV IRES-mediated translation initiation is driven by the high-affinity interaction of the structured IRES element in the 5′ UTR of the viral mRNA with the 40S subunit (Kieft et al., 2001; Otto and Puglisi, 2004). This promotes stable binding of eIF3 and the $\text{eIF2}/\text{GTP}/\text{Met-tRN}{\text{A}}_{\text{i}}^{\text{Met}}$ ternary complex to form a 48S particle with already established codon–anticodon base pairing in the ribosomal P site (Ji et al., 2004; Kolupaeva et al., 1998; Otto and Puglisi, 2004). Similar to the canonical pathway, assembly of active 80S ribosomes still requires eIF5, eIF5B, GTP and 60S subunits (Pestova et al., 1998), but in addition also involves functional interactions of the HCV IRES with the 40S subunit (Locker et al., 2007; Pestova et al., 2008).

In this review, I describe our current structural knowledge of RNA elements found in HCV and HCV-like IRES RNAs and how they are proposed to function in IRES-mediated translation initiation.

## 2 Conserved secondary structure elements in HCV and HCV-like IRES RNAs

**Fig. 1:**
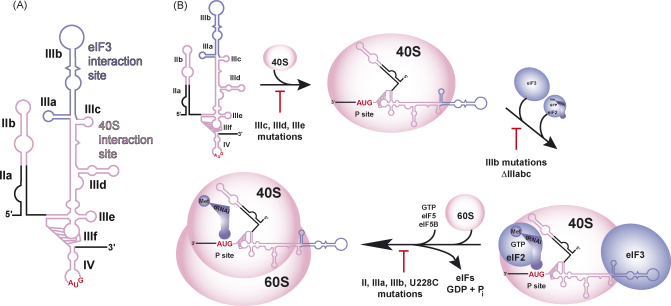
The pathway of HCV IRES-mediated translation initiation. (A) Secondary structure of the HCV IRES RNA with individual domains (II–IV) indicated. The 40S interaction site is shown in pink, the eIF3 interaction site in blue, and the AUG start codon in red. (B) Model of HCV IRES translation initiation. The HCV IRES first binds 40S subunits, then recruits eIF3 and the ternary complex to form a 48S complex. Subsequent 80S formation depends on GTP hydrolysis, eIF5 and eIF5B and requires IRES domains II and apical parts of domain III. Adapted from ref. (Otto and Puglisi, 2004). The 40S subunit is in pink, eIF3 and the ternary complex in blue and mutations affecting specific steps in the assembly are indicated under the arrows.

The HCV IRES displays a secondary structure with two major domains, II and III (Brown et al., 1992; Kieft et al., 1999), which contain all the structural elements crucial for initiation of translation (Fig. 1A) (Ji et al., 2004; Kieft et al., 2001; Otto and Puglisi, 2004; Pestova et al., 1998). The overall domain organisation (II–IV) and several RNA structural motifs in these domains are conserved among related viruses from the *Flaviviridae* family, such as the classical swine fever virus (CSFV), the bovine viral diarrhea virus (BVDV), and GB virus B (GBV-B) (Honda et al., 1999; Pestova and Hellen, 1999; Pestova et al., 1998). This distinct domain organisation has also been found in several members of the *Picornaviridae* family, such as porcine teschovirus (PTV), avian encephalitis virus (AEV), or simian Picornavirus (SPV) suggesting an HCV-like mechanism of translation initiation (Bakhshesh et al., 2008; Hellen and de Breyne, 2007; Pisarev et al., 2004). The 5′ and 3′ boundaries of the HCV IRES have been carefully mapped using dicistronic reporter assays and the IRES element spans from residues 40 through 372 of the viral genome and therefore extends from the 5′ UTR into the ORF (AUG start codon = 342–344) (Fletcher et al., 2002; Fukushi et al., 1994; Honda et al., 1996b; Reynolds et al., 1996, 1995; Rijnbrand et al., 1995).

The larger domain III consists of branching hairpin stem–loops (IIIabcdef) organised in 3- and 4-way junctions (Fig. 1A) (Brown et al., 1992). The basal part of domain III contains a 4-way junction, which includes a predicted pseudoknot (IIIf) and a small stem–loop (IIIe) (Rijnbrand and Lemon, 2000). This region of the IRES displays high conservation of both structural motifs (IIIef) as well as primary sequence (IIIe) and allowed the identification of several HCV-like IRES RNAs from the *Picornaviridae* family (Bakhshesh et al., 2008; Hellen and de Breyne, 2007; Pisarev et al., 2004). The middle part of domain III comprises the conserved stem–loop IIId incorporated into a 3-way (most HCV-like IRESs) or 4-way helical junction (e.g. CSFV) (Brown et al., 1992). Both stem–loops IIIe and IIId also display sequence conservation within the hairpin loop sequence with a 5′-GA(U/C)A-3′ sequence for IIIe and a G-rich hairpin loop sequence with at least 3 consecutive guanosines for domain IIId (Brown et al., 1992; Hellen and de Breyne, 2007). The latter hairpin loop in subdomain IIId can also be accompanied by an internal loop E motif, a common motif found in ribosomal RNA (Correll et al., 1997). The upper part contains a 4-way junction (IIIabc) as found in HCV and CSFV or a 3-way junction lacking the branched hairpin IIIc (e.g. PTV) (Brown et al., 1992; Hellen and de Breyne, 2007). Sequence variability is much more pronounced in this apical part of domain III, but the overall secondary structure is maintained.

The sequences downstream of HCV domain III, surrounding the AUG start codon (IV) have the potential to form a small stem–loop structure, but this feature is not conserved among HCV-like IRESs (Brown et al., 1992; Hellen and de Breyne, 2007; Honda et al., 1996a). The sequences upstream of domain III, namely domain II, also display less conservation than the basal domain III, but predicted structural features, such as an apical hairpin loop and internal loop E motif and basal internal loops are conserved among HCV and closely related HCV-like IRESs (Brown et al., 1992; Hellen and de Breyne, 2007).

## 3 HCV IRES displays distinct 40S subunit and eIF3 interaction sites

The function of the HCV IRES depends on its conserved secondary structure elements as established by extensive mutagenesis of the IRES RNA (Brown et al., 1992; Fukushi et al., 1994; Reynolds et al., 1995; Rijnbrand et al., 1995; Wang et al., 1993, 1994). In solution, these elements fold into an extended tertiary structure with two major, independently folded domains, namely domain III together with its extension domain IV and domain II (Kieft et al., 1999). Domains III and IV contain two major interacting regions: domain IV, the basal domains IIIdef, and domain IIIc bind the 40S ribosomal subunit and the apical domains IIIab provide a platform for eIF3 binding (Kieft et al., 2001; Kieft et al., 1999; Kolupaeva et al., 2000a; Lytle et al., 2001, 2002; Pestova et al., 1998; Sizova et al., 1998) (Fig. 1A). Domain II is organised into a basal domain IIa and an apical domain IIb, of which only domain IIb interacts with the 40S subunit (Honda et al., 1999; Kieft et al., 2001). Ribosomal proteins mediate the IRES–40S subunit interaction as evidenced by 4-thiouridine-mediated UV crosslinking (Otto et al., 2002) and the eIF3 binding region of the IRES directly contacts subunits eIF3a, eIF3b, eIF3d and eIF3f within a binary 40S–eIF3 complex (Buratti et al., 1998; Sizova et al., 1998). Cryo-EM reconstruction of the binary HCV IRES–40S complex showed that interactions of domain III mainly occur in the platform region of the 40S subunit, while domain II contacts the head of the 40S subunit near the E site (Spahn et al., 2001). The cryo-EM structure of the binary HCV IRES–eIF3 complex revealed extensive interactions of this factor within the apical and basal part of domain III of the IRES (Siridechadilok et al., 2005). Further details of these cryo-EM structures have been reviewed recently and are not further discussed in detail (Fraser and Doudna, 2007). The IRES–40S/eIF3 interactions not only provide the binding affinity for 40S subunit and eIF3 recruitment to the HCV IRES, but also function in a coordinated fashion to mediate proper 48S and subsequent 80S ribosome assembly (Ji et al., 2004; Otto and Puglisi, 2004). These various roles of the HCV IRES domains along the initiation pathway and the structures that these functions are encoded in are described in the next section.

### 3.1 Structural motifs of the HCV IRES mediating 40S recruitment

The HCV IRES binds 40S subunits with nanomolar affinity (*K*_d_ = 2 nM) (Kieft et al., 2001; Otto et al., 2002). Neither deletion of domains II and IV, which interact with the 40S subunit nor deletion of domain IIIb (part of eIF3 binding site) affect the binding affinity (Kieft et al., 2001; Otto and Puglisi, 2004). Further deletion of the entire IIIabc junction severely affects 40S binding (*K*_d_ > 500 nM), but only mutations in IIIc reduce 40S binding (up to 10-fold), while changes in IIIa do not (Kieft et al., 2001). This defines domains IIIcdef as the core 40S recruitment domain (Fig. 1B).

**Fig. 2:**
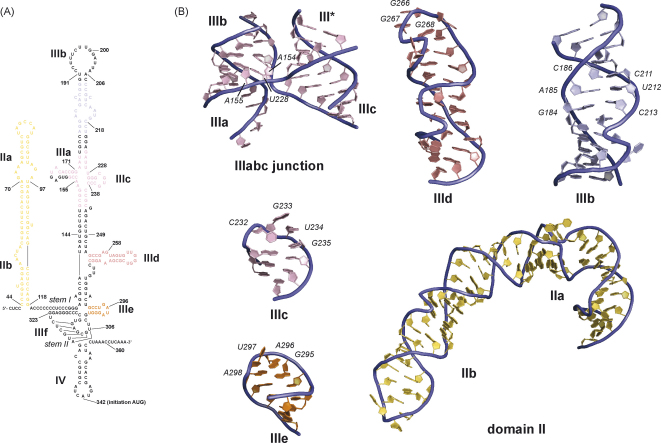
Structures of functional HCV IRES domains. (A) Secondary structure of the HCV IRES with numbering according to Pestova et al. (1998). Nucleotides corresponding to subdomains of unknown structure are in black while nucleotides corresponding to known subdomains determined by NMR or X-ray crystallography are shown in color as follows: yellow for domain II, orange for domain IIIe, light red for domain IIId, pink for junction IIIabc, and light blue for IIIb, respectively. Stems I and II of the pseudoknot are indicated. (B) Structures of HCV IRES domains. The backbone is in a ribbon presentation and colored in blue, while the sugar and bases are colored according to (A). Only the nucleotides corresponding to parts of the HCV IRES are shown, while engineered nucleotides to permit proper folding and stability for structural studies are omitted. Numbering according to (A). The PDB codes of the HCV IRES domains are 1P5P (II), 1F85 (IIIe), 1F84 (IIId), 1IDV (IIIc), 1KH6 (IIIabc junction), and 1KP7 (IIIb), respectively.

The basal part of the 40S binding domain contains a conserved pseudoknot (IIIef), which is crucial for IRES activity (Wang et al., 1995). Evidence for pseudoknot formation is derived from hydroxyl radical probing, which revealed magnesium ion-induced backbone protections in the IIIef 4-way helical junction (Kieft et al., 1999). Chemical and enzymatic probing experiments, on the other hand, suggested an equilibrium with a stem–loop structure, where stem II of the pseudoknot is not formed (Fig. 2A) (Wang et al., 1995). Moreover, mutations, which disrupt base pairing in stem II abolished IRES activity and could be only partially restored by compensatory mutations (Kieft et al., 2001; Wang et al., 1995) (Fig. 2A). Interestingly, most mutations in domain IIIf, which disrupt the pseudoknot, abolish IRES activity, but show only little effect on 40S subunit binding affinity of the HCV IRES, with the exception of stem I mutations (Kieft et al., 2001; Kolupaeva et al., 2000b). The proposed pseudoknot might therefore perform a different function than providing binding affinity, e.g. positioning the downstream AUG start codon in the ribosomal P site, but its precise role still remains to be determined (Kieft et al., 2001; Pestova et al., 1998). Despite its importance for IRES function, the tertiary structure of the pseudoknot has not been determined probably due to the dynamic nature of this IRES domain in the absence of the 40S subunit.

The basal 4-way junction also contains a small stem–loop domain IIIe, which is crucial for IRES function (Lukavsky et al., 2000) (Fig. 2A). The sequence of the hairpin loop (-GAUA-) does not conform to a standard GNRA tetraloop motif and the structure revealed a novel tetraloop fold with 3 major groove exposed bases (G295–U297) in contrast to 3 minor groove exposed bases found in standard GNRA tetraloops (Heus and Pardi, 1991) (Fig. 2B: IIIe). Chemical probing suggests that this hairpin loop is involved in the IRES–40S interaction and converting the loop into a GNRA tetraloop (U297A mutation) not only strongly reduces translational activity but also binding affinity (*K*_d_ > 50 nM) (Kieft et al., 2001; Lukavsky et al., 2000). This suggests a crucial involvement of this highly conserved structural motif in the formation of the binary 40S–IRES complex.

Similarly, domain IIId also contains a hairpin loop (-U_264_UGGGU_269_-) important for 40S binding (Fig. 2B: IIId). Chemical probing showed strong protection of the conserved guanine bases (G266–268) upon 40S binding and mutations of these residues strongly affect both binding affinity and IRES activity (Jubin et al., 2000; Kieft et al., 2001, 1999; Kolupaeva et al., 2000a; Lukavsky et al., 2000). The structure of domain IIId revealed a dynamic hairpin loop with two bases (G266 and G267) exposed to the minor groove and third base (G268) positioned in the major groove as well as rather disordered uracil residues (Klinck et al., 2000; Lukavsky et al., 2000). The internal loop of domain IIId adopts a eukaryotic loop E motif (Correll et al., 1997) with minor groove exposed adenine bases and a characteristic reversal of the backbone direction, a so-called S-turn (Fig. 2B: IIId). In addition, a second S-turn is found in the hairpin loop, where it is introduced by the specific stacking interactions of loop bases (Lukavsky et al., 2000). Both S-turns are located on the same side of domain IIId and could thereby create unique backbone features for the interaction of the HCV IRES with the 40S subunit (Fig. 2B: IIId). This interaction surface seems to be specific for the HCV IRES RNA, since the loop E motif in IIId is not conserved in related HCV-like IRESs, in contrast to the strict conservation of at least three consecutive guanosines in the hairpin loop of domain IIId found in all in HCV and HCV-like IRESs (Hellen and de Breyne, 2007). Domain IIId is commonly incorporated into a 3-way helical junction (or 4-way junction in some HCV-like IRESs) connecting the basal pseudoknot domain with the apical IIIabc 4-way helical junction, but the relative orientations of the helices in this region of the IRES is yet unknown (Fig. 2A) (Hellen and de Breyne, 2007).

The 40S interaction surface of the HCV IRES extends into the IIIabc 4-way helical junction by including the short stem–loop IIIc (Kieft et al., 2001). The structure of domain IIIc also revealed a novel tetraloop fold with all four loop nucleotides exposed to the minor groove (Rijnbrand et al., 2004) (Fig. 2B: IIIc). Converting the sequence to a standard GNRA tetraloop once again abolishes translational activity and reduces 40S affinity 10-fold indicating that both loop fold and sequence are important for IRES activity (Kieft et al., 2001; Rijnbrand et al., 2004; Tang et al., 1999). The 40S interaction surface of the HCV IRES therefore contains three crucial stem–loop structures (IIIc, IIId and IIIe), which all contribute to the high-affinity interaction with the 40S ribosomal subunit. The specific interactions of these domains together with the pseudoknot domain (IIIf) do not just simply mediate 40S subunit recruitment, but also ensure formation of a functional binary complex with the AUG start codon already placed in or near the ribosomal P site to allow subsequent assembly of 48S complexes (Pestova et al., 1998).

### 3.2 Structural motifs of the HCV IRES mediating eIF3 recruitment

After binary 40S–IRES complex formation, both eIF3 and the ternary complex are recruited to form a 48S complex with codon–anticodon base pairing in the P site (Fig. 1B) (Otto and Puglisi, 2004). Binding of eIF3 promotes stable ternary complex binding and is therefore crucial for $\text{Met-tRNA}{\text{i}}_{\text{i}}^{\text{Met}}$ recruitment and 48S complex formation (Fig. 1B) (Ji et al., 2004; Otto and Puglisi, 2004). The binding of eIF3 depends on the intact 4-way helical junction IIIabc (Fig. 1A). The entire HCV IRES as well as the isolated domain IIIabc display the same affinity for the eIF3 complex (*K*_d_ = 35 nM), which is about 15-fold lower as compared to the 40S subunit (Kieft et al., 2001; Sizova et al., 1998). Deletion of domains IIIabc or domain IIIb alone strongly reduces eIF3 binding and stalls translation initiation at the binary 40S–IRES complex stage (Fig. 1B) (Ji et al., 2004; Otto and Puglisi, 2004).

The crystal structure of the IIIabc junction revealed formation of a distorted stack by helices IIIa and IIIb, while IIIc and the helix preceding the junction (III*) stack almost perfectly coaxially (Fig. 2B: IIIabc junction) (Kieft et al., 2002). Mutations, which disrupt hydrogen bonding interactions maintaining the junction fold, such as U228C or A154G and A155G, are deleterious to IRES function indicating that maintenance of the junction fold is crucial for IRES activity (Kieft et al., 2002). The widened minor groove in the distorted helical stack IIIa–IIIb might provide a specific recognition element for the IRES–eIF3 interaction (Kieft et al., 2001). In the crystal, the relative orientation of the helical stacks positions the hairpin loops IIIa and IIIc on the same side of the junction, while IIIb and III* reside on the opposite side (Fig. 2B: IIIabc junction), but time-resolved FRET studies showed that both antiparallel and parallel orientations are sampled in solution (Kieft et al., 2002; Melcher et al., 2003). This suggests a rather dynamic module of the IRES, which might adopt its stable conformation only when bound to the 40S subunit and eIF3.

Other information on the structural organisation of the eIF3 binding domain originates from structures of the internal loop IIIb of the HCV IRES (Fig. 2B: IIIb). This motif contains an intrahelical C186–C211 mismatch, followed by two absolutely conserved Watson–Crick base pairs (A185–U212 and G184–C213), and a mismatch region, which is quite variable in primary sequence between different isolates, but maintains the structural conservation of an S-turn motif (Collier et al., 2002) (Fig. 1B: IIIb). These structural findings combined with results from extensive mutagenesis experiments suggested that backbone features rather than the identity of individual bases are recognized by eIF3 in this region (Collier et al., 2002). The efficient eIF3 recruitment to the binary 40S–IRES complex therefore depends on interactions within the properly folded IIIabc junction and the stem–loop IIIb and is crucial for the stable binding of the ternary complex to form functional 48S complexes.

### 3.3 Structural motifs of the HCV IRES mediating 80S formation

Most mutations within domain III affect either binding of the 40S subunit or eIF3 complex, but the apical domains (IIIabc) might also contain elements crucial for subsequent 80S assembly: deletion of domain IIIb, mutation of the IIIa hairpin loop or mutation in the IIIabc junction (U228C) all exhibit strongly reduced 80S formation (Ji et al., 2004; Otto and Puglisi, 2004). The reduced IRES activity of these mutants might simply reflect the lowered eIF3 or 40S subunit binding affinity and resulting less efficient binary or 48S complex formation, but yet unknown roles during subunit joining cannot be excluded (Ji et al., 2004). Domain II, in contrast, is not required for 40S subunit binding (Kieft et al., 2001; Otto et al., 2002) and its deletion does not alter eIF3 and ternary complex recruitment, but reduces the translational activity up to 5-fold by blocking 80S formation (Ji et al., 2004; Locker et al., 2007; Otto and Puglisi, 2004).

The cryo-EM analyses of the 40S subunit and binary HCV IRES–40S complexes revealed significant, domain II-dependent conformational changes in the 40S subunit upon IRES binding (Spahn et al., 2001). The interaction of the apical domain IIb near the E-site leads to a rotation of the ribosomal head relative to the body and the resulting opening of the mRNA entry channel latch aids to stably accommodate the HCV ORF (Kolupaeva et al., 2000a; Spahn et al., 2001). The main role of the domain II-dependent conformational changes of the 40S subunit however is downstream of 48S assembly, in stimulating hydrolysis of eIF2-bound GTP and subsequent eIF2 release during subunit joining and this function is conserved among HCV-like IRESs (Locker et al., 2007). In addition, domain II also seems to play a direct role in subunit joining, since 48S complexes assembled onto CSFV IRES in the absence of eIF2 still require domain II for efficient subunit joining suggesting a general role of domain II in 80S assembly and eIF-release (Locker et al., 2007; Pestova et al., 2008).

The structure of the entire domain II has been determined by NMR spectroscopy (Lukavsky et al., 2003). Domain II forms an independently folded module within the IRES RNA with an overall L-shape both free in solution and when bound to the 40S subunit (Fig. 2B: domain II) (Kim et al., 2002; Lukavsky et al., 2003; Spahn et al., 2001). The characteristic 90 degree bend in the basal domain IIa is introduced by the stacking interactions of five single stranded bases (-A_53_ACUA_57_-) and stabilised by magnesium ion binding in two locations in the major groove (Dibrov et al., 2007; Lukavsky et al., 2003). The primary sequence of domain IIa in closely related HCV-like IRESs differs considerably, but the bend is a conserved feature and its deletion results in five-fold reduction in translational activity, the same as deletion of the entire domain II (Locker et al., 2007). The conserved L-shape of domain II might help to place the apical domain IIb into the mRNA exit site on the 40S subunit (Spahn et al., 2001), where it interacts with the ribosomal head protein S5 (rpS5), and alters 40S subunit conformation (Fukushi et al., 2001; Kolupaeva et al., 2000a; Spahn et al., 2001). Domain IIb contains a loop E motif and a highly conserved hairpin loop (-U_80_AGCCAU_86_-) with a dynamic, looped-out uracil on the 3′ side (Lukavsky et al., 2003). These features are reminiscent of domain IIId, but in IIb the looped-out uracil and the S-turn are located on opposite sides of the domain, while they are found both on the 3′ side in domain IIId (Lukavsky et al., 2000). This creates very different 40S interaction surfaces in both subdomains despite using very similar RNA modules. Substituting the hairpin loop by a stable UNCG tetraloop or the loop E motif by Watson–Crick base pairs also reduces translation activity by 80%, since these mutations block eIF2 release and subsequent subunit joining (Locker et al., 2007). Interestingly, the specific function of domain II lacks a counterpart in the canonical cap-dependent initiation pathway, where start codon recognition is believed to induce conformational changes that stimulate hydrolysis of eIF2-bound GTP and trigger *P*_i_ release, thereby committing the 48S complex into 80S assembly (Algire et al., 2005; Unbehaun et al., 2004). Whether domain II-dependent conformational changes of the 40S subunit are similar to the ones induced by AUG start codon recognition is unknown.

## 4 Conclusion

Research over the past decade has shown that IRES function is governed by structure. We now know the 3D structures of most HCV IRES subdomains mediating 40S subunit and eIF3 recruitment as well as subunit joining, but we still lack an overall structure of the HCV or an HCV-like IRES and any atomic details of their interaction with the ribosome or eIF3. The low-resolution EM models of the binary HCV IRES–40S, 80S and eIF3 complexes need to be followed up by atomic resolution models of these assemblies. Furthermore cryo-EM studies of more complex assemblies up to elongating ribosomes are required in order to understand the structural basis of how the HCV IRES manipulates the translational machinery and how it controls the different stages of translation initiation by modulating 40S subunit and eIF conformations. Knowledge of these details will not only enrich our understanding of the eukaryotic ribosome, but will also aid structure-based drug design to develop antiviral treatments in the future.
